# Comparison of [^18^F]-FDOPA PET and [^123^I]-FP-CIT SPECT acquired in clinical practice for assessing nigrostriatal degeneration in patients with a clinically uncertain parkinsonian syndrome

**DOI:** 10.1186/s13550-022-00943-6

**Published:** 2022-10-22

**Authors:** Elon Wallert, Erwann Letort, Friso van der Zant, Ania Winogrodzka, Henk Berendse, Martijn Beudel, Rob de Bie, Jan Booij, Pieter Raijmakers, Elsmarieke van de Giessen

**Affiliations:** 1 Department of Radiology & Nuclear Medicine, Amsterdam UMC, Location University of Amsterdam, Meibergdreef 9, 1105 AZ Amsterdam, The Netherlands; 2 Department of Nuclear Medicine, Northwest Clinics, Location Alkmaar, Wilhelminalaan 12, Alkmaar, The Netherlands; 3 Department of Radiology & Nuclear Medicine, Spaarne Gasthuis, Location Haarlem, Boerhaavelaan 22, Haarlem, The Netherlands; 4 Department of Neurology, Northwest Clinics, Location Alkmaar, Wilhelminalaan 12, Alkmaar, The Netherlands; 5 Department of Neurology, Amsterdam UMC, Location Vrije Universiteit Amsterdam, Boelelaan 1117, Amsterdam, The Netherlands; 6 Department of Neurology, Amsterdam UMC, Location University of Amsterdam, Meibergdreef 9, Amsterdam, The Netherlands; 7 Department of Radiology & Nuclear Medicine, Amsterdam UMC, Location Vrije Universiteit Amsterdam, Boelelaan 1117, Amsterdam, The Netherlands

**Keywords:** ^18^F-FDOPA, PET, Dopamine transporter, ^123^I-FP-CIT SPECT, Nigrostriatal degeneration, Parkinsonism

## Abstract

**Purpose:**

Two commonly used imaging techniques to aid in the diagnosis of neurodegenerative parkinsonian syndromes are dopamine transporter (DAT) imaging with [^123^I]-FP-CIT single-photon emission computed tomography (DAT-SPECT) and positron emission tomography with [^18^F]-FDOPA (FDOPA-PET). This paper provides a unique series of parkinsonian patients who received both FDOPA-PET and DAT-SPECT in routine clinical practice and compares the reported results to assess potential differences between these two imaging techniques.

**Methods:**

We present 11 patients with a clinically uncertain parkinsonian syndrome (CUPS), who received both FDOPA-PET and DAT-SPECT. All patients received an FDOPA-PET scan and DAT-SPECT as part of routine clinical care.

**Results:**

The median time between the F-DOPA-PET scan and DAT-SPECT scan was 6 months (range 0–15 months). There was a discrepancy in the reported results of the FDOPA-PET and DAT-SPECT scans in nine patients, including 7 patients whose FDOPA-PET scan was reportedly normal, whereas their DAT-SPECT scan was abnormal.

**Conclusions:**

In this case series of CUPS patients, DAT-SPECT was more often rated as abnormal than FDOPA-PET. The striatal loss of FDOPA uptake can be less pronounced than that of DAT binding in CUPS patients in early disease stages. Consequently, the interpretation of FDOPA-PET scans in CUPS can sometimes be challenging in routine practice.

**Supplementary Information:**

The online version contains supplementary material available at 10.1186/s13550-022-00943-6.

## Introduction

The clinical diagnosis of Parkinson’s disease (PD) by a movement disorder specialist is usually rather straightforward. However, in early-stage PD one or more of the cardinal motor symptoms might be absent or can be subtle. The available literature reports an accuracy of the clinical diagnosis of 80% in early-stage PD [1]. To aid in the diagnosis in clinically uncertain parkinsonian syndromes (CUPS), [^18^F]-DOPA positron emission tomography (FDOPA-PET) and dopamine transporter (DAT) imaging using [^123^I]-FP-CIT single-photon emission computed tomography (further referred to as DAT-SPECT) scans can be used to visualize nigrostriatal dopaminergic neurodegeneration, a neuropathological hallmark of PD.

In the synthesis of dopamine, decarboxylation by aromatic L-amino acid decarboxylase (AADC) catalyzes the conversion of L-dopa to dopamine. [^18^F]-DOPA is, similar to L-dopa, a substrate for AADC and hence a measure of the decarboxylation process. [^123^I]-FP-CIT binds with high-affinity to the dopamine transporter (DAT), which is expressed in the presynaptic neuron and enables reuptake of dopamine from the synaptic cleft. In case of dopaminergic neurodegeneration in PD, both the striatal FDOPA uptake and the DAT binding are reduced [2].

There is limited data comparing FDOPA-PET and DAT-SPECT for detecting dopaminergic neurodegeneration. A recent systematic review and meta-analysis concluded that the decrease in activity in the striatum of PD patients is consistently smaller in studies assessing FDOPA-PET scans compared with DAT-SPECT scans [2]. In only three studies, both scan types were used in the same individuals. These articles did not report significant group differences, but the focus was not on CUPS and imaging was performed in a research setting [3–5].

The aim of this paper is to provide a unique series of clinical cases of patients with a CUPS who received both FDOPA-PET and DAT-SPECT in clinical practice to highlight potential differences between these techniques.

## Case series

**Fig. 1:**
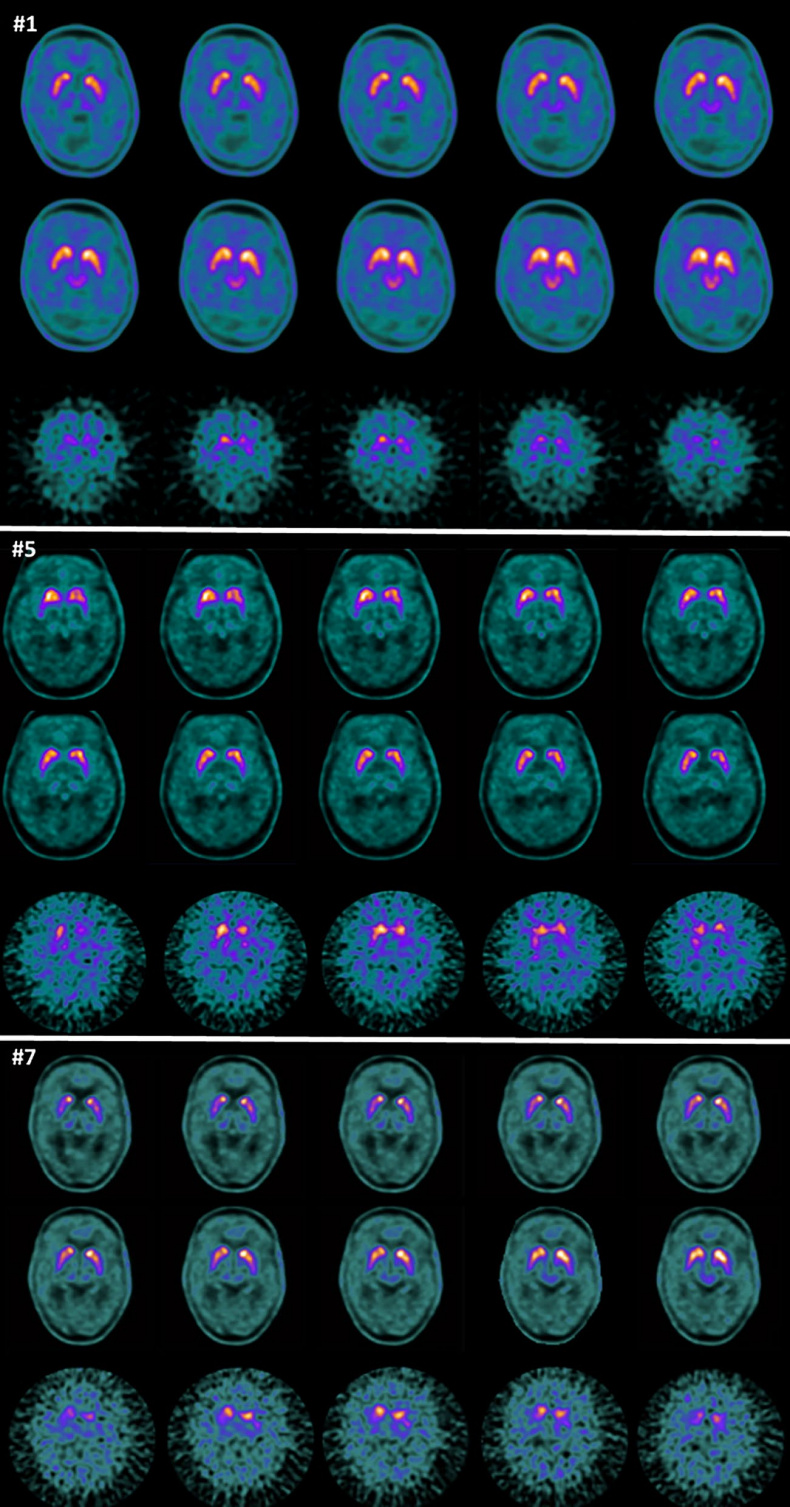
Representative transverse images of the striatum on FDOPA-PET (2 top rows for each case) and DAT-SPECT (bottom row for each case) for patients #1, #5 and #7. For all three cases, FDOPA-PET was reported normal and DAT SPECT abnormal

We retrospectively identified a consecutive series of patients, who had received both FDOPA-PET and DAT-SPECT scans since 2015 (Table 1). All patients in this case series visited a neurologist at a movement disorder clinic for parkinsonian symptoms and received an FDOPA-PET scan as part of routine clinical care, since a clinical diagnosis could not be established with sufficient probability (Northwest Clinics, location Alkmaar). MRI scans were acquired in most patients to exclude structural changes as cause of the parkinsonian symptoms. (Patients 1 and 10 did not receive an MRI prior to FDOPA-PET scans.) In the eleven patients presented in this case series, the diagnosis remained uncertain after the FDOPA-PET scan. Therefore, three patients received an additional DAT-SPECT at Northwest Clinics, location Alkmaar, and eight patients were referred to a tertiary university hospital (Amsterdam UMC), where patients were seen by a neurologist and an additional DAT-SPECT scan was performed (six at Amsterdam UMC, location Vrije Universiteit, two at location University of Amsterdam; Fig. 1 and Additional file 1: Fig. S2).

**Table 1:** Case overview with clinical and imaging information

	Symptoms	Hoehn and Yahr	Age	Disease duration in years	History of psychiatric or neurologic disorders	Result FDOPA-PET Reported uptake	Result DAT-SPECT Reported binding	FDOPA-PET				DAT-SPECT				Months between scans	Final diagnosis
								CR	CL	PR	PL	CR	CL	PR	PL		
Patient 1^1^	Restless while sleeping, dysarthria, weakness, cognitive symptoms	N/A	68	0.75	–	Normal	Severely reduced	2.47	2.56	2.29	2.33	1.58	1.47	1.82	1.78	2	Possible MSA-P
Patient 2^1^	Walking disorder, tremor, bradykinesia, rigidity	3	51	3	–	Slightly reduced	Severely reduced	1.59	1.70	1.69	1.67	2.26	2.13	1.88	1.83	3	PD
Patient 3^1^	tremor	2	66	1	Epilepsy	Normal	Reduced	2.08	2.12	2.11	2.06	2.46	2.44	1.99	1.88	< 1	PD
Patient 4^2^	Pain, mild rigidity, tremor	N/A	65	0.75	–	Normal	Normal	2.35	2.59	2.96	2.93	3.45	3.4	3.33	3.24	7	Inconclusive
Patient 5^2^	Urine incontinence, walking disorder, dysarthria, bradykinesia, rigidity	N/A	60	8	–	Normal	Reduced	2.43	2.46	2.71	2.49	2.2	1.9	2	1.9	11	Probable MSA-P
Patient 6^2^	Walking disorder, tremor, rigidity	3	70	3	Epilepsy	Normal	Severely reduced	2.37	2.49	2.25	2.33	2.5	2.6	1.6	1.8	7	PD
Patient 7^2^	Walking disorder, bradykinesia	3	77	3.5	vitamin B12 deficiency	Normal	Reduced	1.99	2.52	2.38	2.35	2.5	2.0	1.9	2.1	6	PD
Patient 8^2^	Walking disorder, dysarthria, mask expression, bradyphrenia, bradykinesia,	N/A	69	0.7	–	Normal	Normal	2.57	2.68	2.98	2.86	2.97	2.87	2.62	2.45	3	Inconclusive (passed away)
Patient 9^3^	tremor	2	76	1	transient bilateral ptosis (side effect)	Asymmetric slightly reduced	Reduced	2.10	2.20	2.37	2.29	2.47	2.37	2.28	2.03	9	PD
Patient 10^3^	Tremor, mask expression	1	67	2	–	Normal	Reduced	2.92	2.89	2.50	2.52	1.69	1.68	1.31	1.16	15	PD
Patient 11^2^	Tremor, bradykinesia, mask expression	2	65	1	–	Normal	Severely reduced	2.55	2.61	2.11	2.03	1.9	1.7	1.5	1.4	7	PD

1 = DAT-SPECT performed at Northwest Clinics, 2 = DAT-SPECT performed at Amsterdam UMC, location Vrije Universiteit, 3 = DAT-SPECT performed at Amsterdam UMC, location University of Amsterdam, CR = right caudate nucleus-to-occipital cortex ratio, CL = left caudate nucleus-to-occipital cortex ratio, PR = right putamen-to-occipital cortex ratio, PL = left putamen-to-occipital cortex ratio

Acquisition of FDOPA-PET and DAT-SPECT scans was performed according to international guidelines (Additional file 1). None of the patients used dopaminergic medication at the time of the FDOPA-PET scan. FDOPA-PET and DAT-SPECT scans were assessed visually by experienced nuclear medicine physicians (5–25-year experience) and semiquantitatively, as the ratio of counts in the region of interest (caudate nucleus and putamen) divided by the counts in the reference region (occipital cortex) (Additional file 1: Fig. S1). At Amsterdam UMC, the semiquantitative results for DAT-SPECT were compared to locally acquired databases of age-matched healthy controls. (Ratios > 2 standard deviations below age-matched controls were considered abnormal.)

The median age of the patients was 66.7 years (range 51–77 years) and the mean disease duration 2.2 years at time of presentation (range 8 months–8 years). The final clinical diagnosis was PD for seven patients, possible or probable MSA-P for two patients, and remained inconclusive, but with no evidence of neurodegeneration, for two patients with normal FDOPA-PET, DAT-SPECT and MR imaging. The median time between the F-DOPA-PET scan and DAT-SPECT scan was 6 months (range 0–15 months).

The striatal uptake in the FDOPA-PET scans was reported as normal for nine patients and slightly reduced for two patients. In the two patients that had reduced uptake on the FDOPA-PET scan, the pattern was described as atypical for PD. In the other patients, a DAT-SPECT scan was acquired if the diagnosis remained uncertain after a reportedly normal FDOPA-PET scan. The reports of the DAT-SPECT scans described normal striatal binding in only two patients, reduced binding in five patients and severely reduced binding in four patients. Thus, there were discrepancies between the reported results of the FDOPA-PET and DAT-SPECT scans in nine patients. The visual differences in the striatal uptake on FDOPA-PET and binding on DAT-SPECT were also clearly visible (Fig. 1; Additional file 1: Fig. S2).

## Discussion

In this case series, we show that in a subset of patients with CUPS, there is a clear discrepancy in clinical reporting of normality versus abnormality between FDOPA-PET and DAT-SPECT. Given the short time interval between the acquisition of both scans, discrepancies cannot be explained by disease progression (only). For example, the annual reduction in striatal DAT binding in PD patients is about 5% and visually difficult to detect [6]. However, there is a clearly visible difference between the striatal uptake on FDOPA-PET and uptake on DAT-SPECT (Fig. 1; Additional file 1: Fig. S2).

A systematic review with meta-analysis that compared DAT tracers and FDOPA found a consistently smaller reduction in striatal uptake of FDOPA compared with the DAT tracers [2] in line with our results. However, this systematic review did not report on articles that directly compared FDOPA-PET and DAT-SPECT within the same subject. Three studies were identified that reported on a direct comparison. Two studies found no significant differences in sensitivity and specificity for both techniques [3, 5]. The third study included twenty patients with PD and found that in three patients the DAT-SPECT scan showed more reduced striatal binding than the FDOPA-PET scan. However, in two out of three patients the FDOPA-PET scan was a better reflection of the clinical symptoms [4]. In addition, there is one PET study comparing FDOPA with [^11^C]methylphenidate (a DAT tracer), which concluded that the reduction of striatal DAT binding is larger in early PD than the reduction of AADC activity [7]. It should be noted that these studies included patients with an established clinical diagnosis of PD and did not include patients with CUPS. Taken together, the literature consistently points to more outspoken reductions in striatal binding on DAT-SPECT than FDOPA-PET. This is reflected as well in the qualification of DAT imaging as an enrichment biomarker for clinical trials targeting early stages of PD by the European Medicines Agency [8]. However, it is likely that differences between DAT and AADC imaging in the majority of cases usually will not impact clinical practice.

A possible explanation for the differences between FDOPA-PET and DAT-SPECT scans is compensatory changes in the striatal dopaminergic nerve terminals in patients with early PD. To compensate for the dopaminergic cell loss, striatal AADC activity may be upregulated in surviving neurons to produce more dopamine, whereas DAT expression may be downregulated to delay uptake of dopamine from the synapse to prolong transmission of the dopaminergic signal [2, 3, 7, 9].

In addition, the smaller reductions in striatal uptake of FDOPA compared with the DAT tracers might result in more subtle visual abnormalities on FDOPA-PET than on DAT-SPECT scans. Hence, FDOPA-PET might be more prone to misinterpretation and inter-rater variability; indeed, some of the presented FDOPA-PET scans that were originally reported as normal could retrospectively be considered as abnormal. Discrepancies in the interpretation of FDOPA-PET scans for dopaminergic deficit syndromes between readers were reported previously [10]. Also, criteria for the visual analysis of DAT-SPECT scans are well established and have been compared with the combined results of visual analyses with quantitative results [11, 12]. However, criteria for a visual analysis of FDOPA-PET scans (e.g., due to the better spatial resolution of PET than SPECT, the highest [^18^F]-FDOPA uptake is in the anterior putamen instead of the caudate nucleus in healthy controls, and in PD there is relatively increased midbrain extra-striatal uptake and caudate sparing [13]) are less widely established, although they have been described in the EANM/SNMMI guidelines for dopaminergic imaging in parkinsonian syndromes [14]. Previous research showed a high diagnostic accuracy of quantitative analysis of FDOPA-PET scans of 93% to differentiate healthy controls from a group of parkinsonian syndromes characterized by dopaminergic degeneration [13]. However, the value of quantitative information as an addition to visual analysis is unknown, particularly in CUPS.

In this article, we report on patients with CUPS in a routine clinical setting. All patients first received an FDOPA-PET scan that was reported to be (borderline) normal. Therefore, only patients that had symptoms that were inconsistent with the result of the FDOPA-PET scan received a DAT-SPECT scan, which may have introduced a bias. Additionally, the DAT SPECT scans were acquired on three different SPECT cameras and reconstructed and analyzed by the software that was used at the location where the DAT SPECT scan was acquired. Hence, the quantitative information can slightly vary per location. Moreover, as this paper reports on clinical practice, the FDOPA-PET and DAT-SPECT scans were reported by different experienced nuclear medicine physicians (5–25-year experience), which might introduce inter-rater variability. Nevertheless, sometimes the interpretation of FDOPA-PET scans can be more challenging than that of DAT-SPECT scans in routine practice in CUPS patients. Therefore, consistency in the way scans are acquired, reconstructed, quantified as well as the use of uniform interpretation criteria may increase the diagnostic accuracy of such scans in CUPS.

In conclusion, in patients with a CUPS, visual changes in FDOPA-PET might be more subtle than on DAT-SPECT. Therefore, DAT-SPECT may more often be rated as abnormal than FDOPA-PET in routine clinical practice, warranting increased attention to the use of uniform interpretation criteria. Part may be a reflection of compensatory downregulation of the DAT and possibly upregulation of DOPA decarboxylase activity in early disease stages. Although both FDOPA-PET and DAT-SPECT are well-validated tools to aid the diagnosis of PD or atypical parkinsonism, it is good to be aware of the differences between these imaging methods, particularly in patients with CUPS.

## Supplementary Information

**Additional file 1**. Supplemental material includes supplementary methods and supplementary figure S1 and S2.

## Data Availability

All data generated or analyzed during this study are included in this published article [and its supplementary information files].
