# Supplementary material for: Comparison of [18F]-FDOPA PET and [123I]-FP-CIT SPECT acquired in clinical practice for assessing nigrostriatal degeneration in patients with a clinically uncertain parkinsonian syndrome
Source: EJNMMI Res. 2022 Oct 22;12:68. doi: 10.1186/s13550-022-00943-6 (PMC9588108; doi:10.1186/s13550-022-00943-6)
Supplement: Supplementary file 1 — Additional file 1. Supplemental material includes supplementary methods and supplementary figure S1 and S2. [file 13550_2022_943_MOESM1_ESM.docx]

**Supplementary material**

**Supplementary methods**

*FDOPA PET acquisition*

Patient preparation for FDOPA-PET included an amino acid reduced diet for 12 hours and no food intake for 6 hours prior to the scan. One hour after oral administration of 200 mg carbidopa, ~200 MBq [^18^F]-FDOPA was injected intravenously followed by a 10 minute PET acquisition of the brain at 90 minutes post-injection on a Siemens Biograph-16 TruePoint PET/CT system (Siemens Healthcare, Knoxville, Tennessee, USA). A low-dose CT of the brain was acquired for attenuation correction. The head of the patient was positioned along the orbitomeatal line. Images were reconstructed using TrueX with Gaussian filter, 5 iterations and 8 subsets. Full width at half maximum was 2 mm and zoom factor 2.2.

Semi quantitative analysis was performed by manually placing regions of interest for the right and left caudate nucleus and putamen and the occipital cortex on the three consecutive slices with the highest striatal uptake in the region ratio software of the Siemens Symbia S SPECT camera system (Siemens, Erlangen, Germany).

*DAT SPECT acquisition*

All subjects did not use any medication known to influence [^123^I]-FP-CIT binding^1^ and were prepared with oral potassium iodide (Northwest Clinics, location Alkmaar and Amsterdam UMC, location University of Amsterdam) or oral potassium chlorate (Amsterdam UMC, location Vrije Universiteit) to block thyroid uptake of free radioactive iodide.

Northwest Clinics, location Alkmaar

SPECT scans were acquired 3 hours^2^ after intravenous injection of ~185 MBq [^123^I]-FP-CIT (GE Healthcare) on a Siemens Symbia S dual-head gamma camera (Siemens Healthcare, Knoxville, Tennessee, USA) with a LEHR collimator. The head of the patient was positioned along the orbitomeatal line. Scan duration was around 45 minutes. Images were reconstructed using filtered back projection with a Butterworth filter (order 10, cut-off 0.55 cycles/cm).

Semi quantitative analysis was performed by manually placing standard regions of interest for the right and left caudate nucleus and putamen and the occipital cortex on the three consecutive slices with the highest striatal uptake in the region ratio software of the Siemens Symbia S SPECT camera system (Siemens, Erlangen, Germany).

Amsterdam UMC, location Vrije Universiteit

The SPECT imaging protocol has been described in detail previously^3^. Three hours after intravenous injection of ~185 MBq [^123^I]-FP-CIT (GE Healthcare)^2^, patients were scanned using a Siemens E.Cam dual-head gamma camera (Siemens, Munich, Germany) with a fan-beam collimator. The head of the patient was positioned along the orbitomeatal line. Scan duration was 30 minutes. Images were reconstructed using filtered back projection with a Butterworth filter (order 8, cut-off 0.6 cycles/cm).

Semi quantitative analysis was performed as described previously^3^: five fixed-size regions of interest for the left and right caudate nucleus, left and right putamen and occipital cortex were manually placed on three adjacent axial SPECT slices. Image analysis was performed with the region ratio software of the E.Cam (Esoft, version Syngo VE2B, Siemens, Erlangen, Germany).

Amsterdam UMC, location University of Amsterdam

The SPECT system and imaging protocol has been described in detail previously^4^. Patients were injected with ~111 MBq [^123^I]-FP-CIT (GE Healthcare) and scanned 3 hours post-injection on an InSPira system, which is a brain-dedicated SPECT system that differs from conventional SPECT scanners with respect to hardware configuration and scanning geometry resulting in increased spatial resolution^4^. The detector ring consists of two clamshells, each containing 12 (focusing) fanbeam collimators. The head of the patient was positioned along the orbitomeatal line. A proprietary iterative reconstruction algorithm (with 60 iterations) was used to reconstruct the data into 3D images. This iterative reconstruction algorithm is based on a maximum a posteriori (MAP) estimation and includes a point spread function (PSF). An adult head CT template was manually aligned and used for attenuation correction.

Semi-quantitative analysis was performed automatically using BRASS (BRASS™, HERMES Medical, Sweden), as described previously^3^. BRASS registers the patient data to a template with volumes of interest for right and left caudate nucleus, putamen, whole striatum and occipital cortex.

**Supplementary results**

*Medication during FDOPA PET and DAT SPECT*

As described in the manuscript none of the patients used dopaminergic medication at the time the FDOPA-PET scan was performed.

Two patients were using levodopa at the time of the DAT SPECT scan. Levodopa was continued during the DAT SPECT in accordance to the European Association of Nuclear Medicine guidelines.^5^ One patient (patient 7) used amitriptyline at the time of the DAT SPECT scan. However, influence of tricyclic antidepressants on DAT imaging is not likely.^1^ No other medication that might influence DAT SPECT outcomes was reported.

**Response to dopaminergic therapy.**

Some of the patients received a trial of dopaminergic therapy. Patient 1 (possible MSA-P) did not show clinical improvement after a trial of dopaminergic therapy. Patients 2 and 3 (PD) had a good clinical response. Patient 5 (probable MSA-P) and patient 9 (inconclusive diagnosis) had clinically limited improvement of symptoms on a low dose of dopaminergic therapy. Patient 9 (PD) showed clinical improvement, however due to side-effects, the trial of dopaminergic therapy was discontinued. For patients 4 (inconclusive), 6, 7, 10, and 11 (PD) information on the clinical response to dopaminergic therapy was not available.

**Supplementary figure S1**
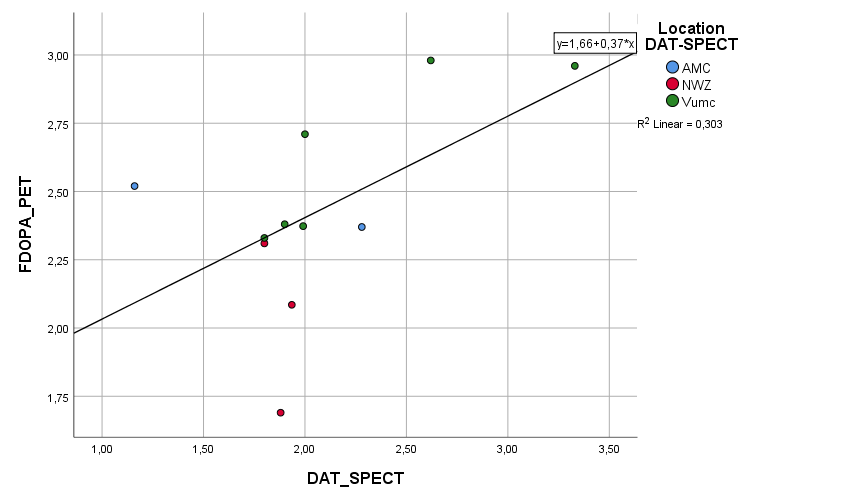


*Correlation of the uptake / binding values of FDOPA-PET and DAT-SPECT of the putamen contralateral to the clinically most affected side of the body. Right putamen for patients 2, 4, 5, 7, 8, and 9. Left putamen for patients 6 and 10. For patients 1, 3, and 11 an average value was taken, since there was not a clearly most affected side of the body. Pearson correlation was 0.549 with a p-value of 0.1. Please note that different SPECT cameras and different collimators were used, which will have influenced the binding ratios for DAT SPECT.*

**Supplementary references**

1. Booij J, Kemp P. Dopamine transporter imaging with [(123)I]FP-CIT SPECT: potential effects of drugs. Eur J Nucl Med Mol Imaging. 2008;35(2):424-38.

2. Booij J, Hemelaar TG, Speelman JD, de Bruin K, Janssen AG, van Royen EA. One-day protocol for imaging of the nigrostriatal dopaminergic pathway in Parkinson's disease by [123I]FPCIT SPECT. J Nucl Med. 1999;40(5):753-61.

1. Vriend C, Raijmakers PGHM, Veltman DJ, van Dijk KD, van der Werf YD, Foncke EMJ, Smit JH, Berendse HW, van den Heuvel OA. Depressive symptoms in Parkinson's disease are related to reduced [^123^I]FP-CIT binding in the caudate nucleus. J Neurol Neurosurg Psychiatry. 2014;85(2):159-64.
2. Adriaanse SM, de Wit TC, Stam M, Verwer E, de Bruin KM, Booij J. Clinical evaluation of [123I]FP-CIT SPECT scans on the novel brain-dedicated InSPira HD SPECT system: a head-to-head comparison. EJNMMI Res. 2018;8:4-9.
3. Morbelli S, Esposito G, Arbizu J, Barthel H, Boellaard R, Bohnen NI, Brooks DJ, Darcourt J, Dickson JC, Douglas D, Drzezga A, Dubroff J, Ekmekcioglu O, Garibotto V, Herscovitch P, Kuo P, Lammertsma A, Pappata S, Peñuelas I, Seibyl J, Semah F, Tossici-Bolt L, Van de Giessen E, Van Laere K, Varrone A, Wanner M, Zubal G, Law I. EANM practice guideline/SNMMI procedure standard for dopaminergic imaging in Parkinsonian syndromes 1.0. Eur J Nucl Med Mol Imaging. 2020;47(8):1885-1912.

**Supplementary figure S2.**

FDOPA-PET (2 top rows) and DAT-SPECT (bottom row) images of patients 2, 3, 4, 6, 8,9, and 10 (see Table 1 for details).

Patient 2


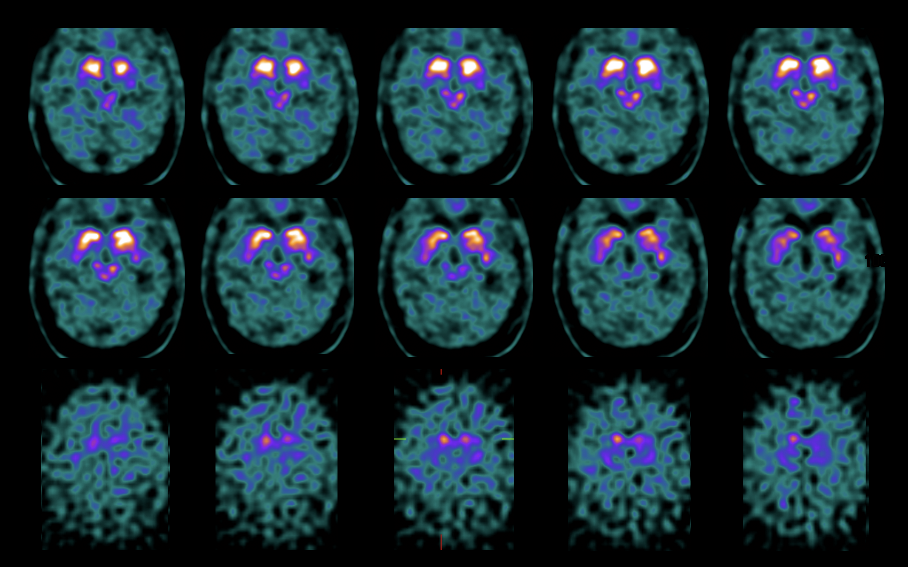


Patient 3


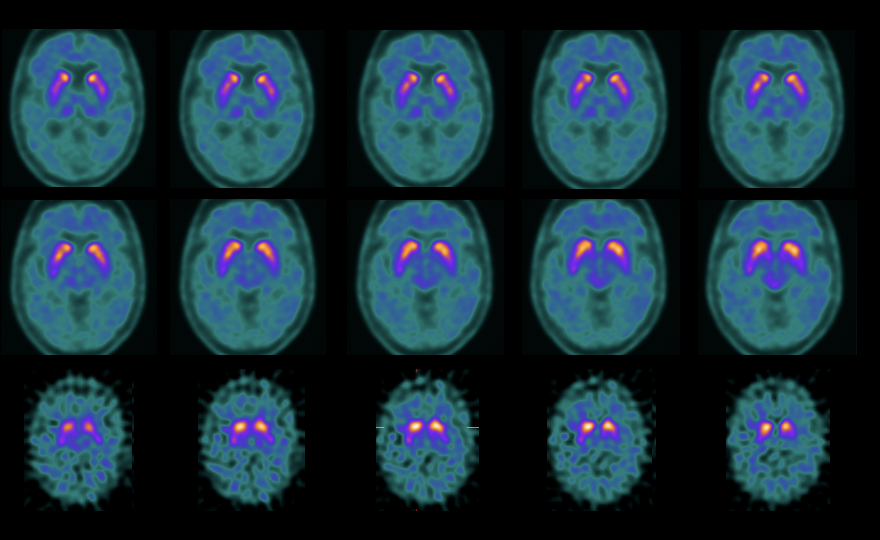


Patient 4


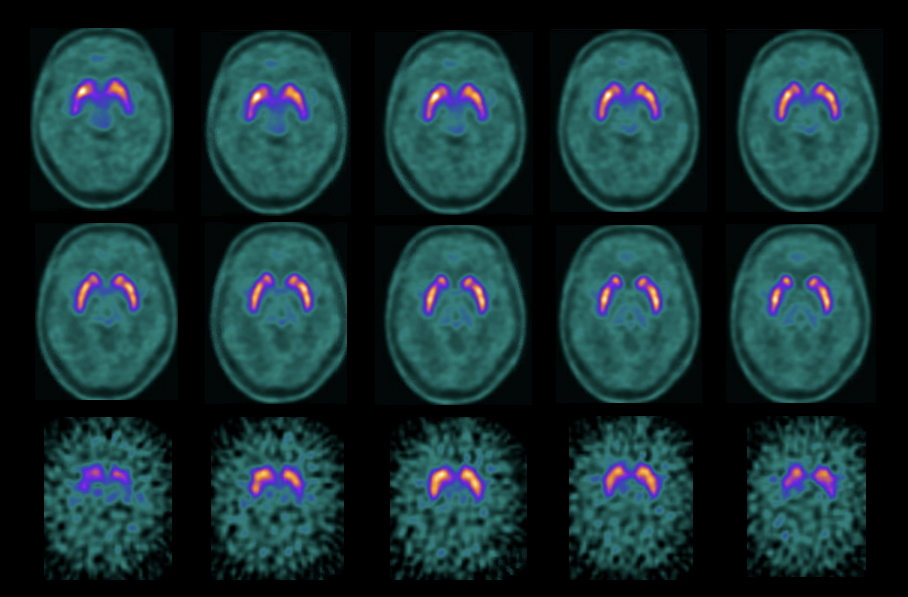


Patient 6


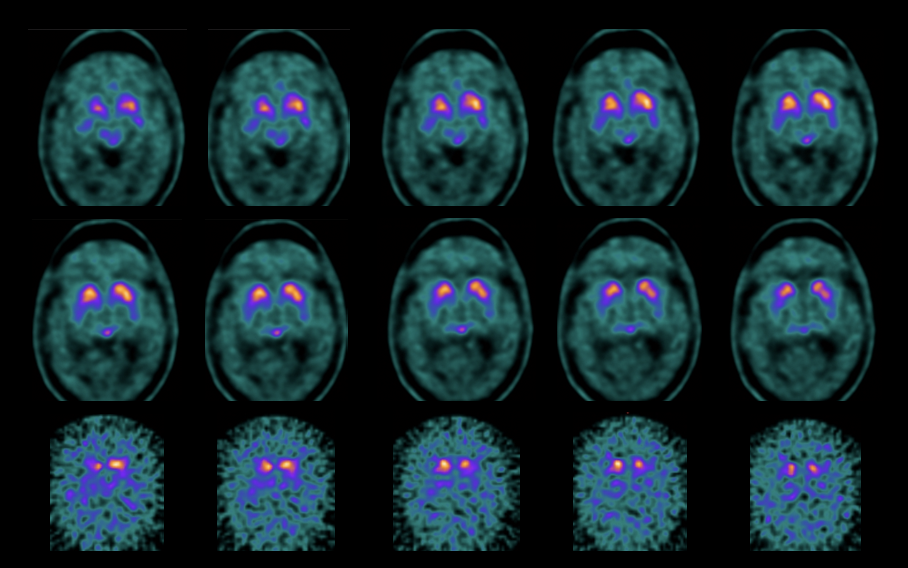


Patient 8


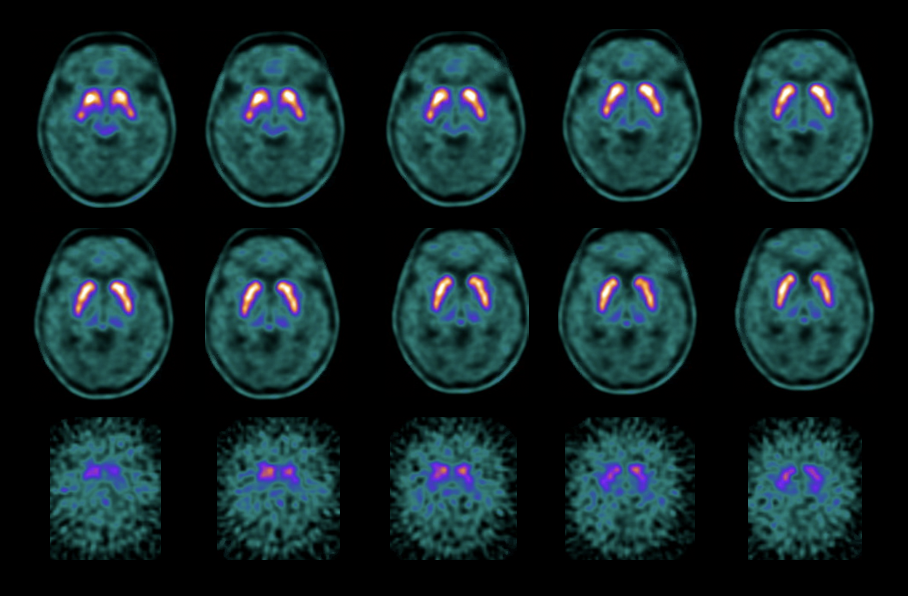


Patient 9


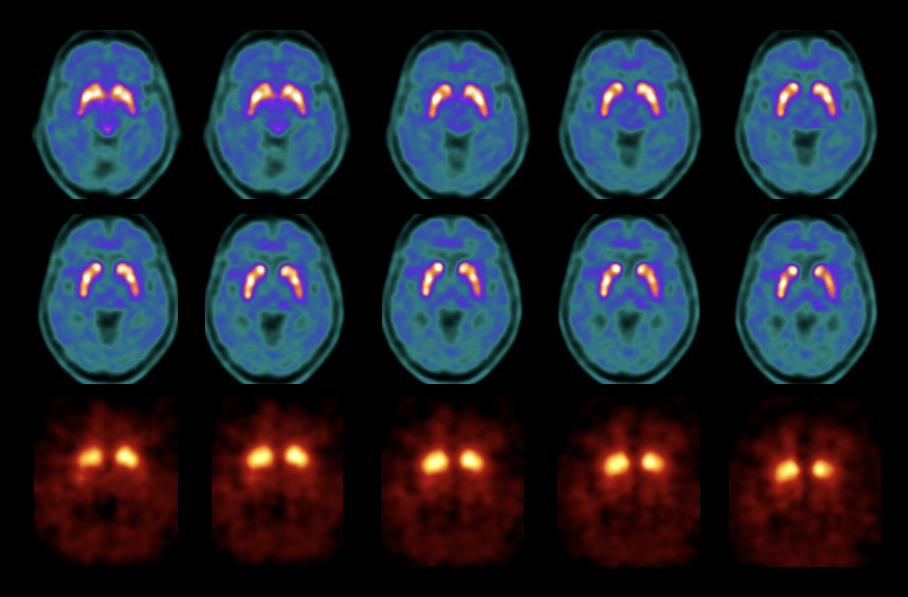


Patient 10


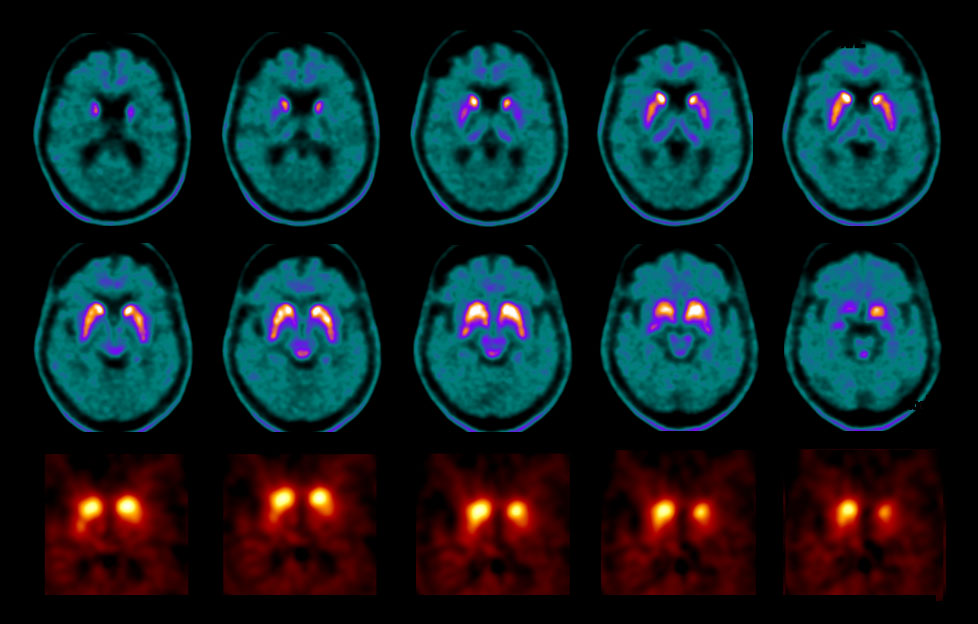


Patient 11
